# Responses of fungal community composition to long‐term chemical and organic fertilization strategies in Chinese Mollisols

**DOI:** 10.1002/mbo3.597

**Published:** 2018-03-23

**Authors:** Mingchao Ma, Xin Jiang, Qingfeng Wang, Marc Ongena, Dan Wei, Jianli Ding, Dawei Guan, Fengming Cao, Baisuo Zhao, Jun Li

**Affiliations:** ^1^ Institute of Agricultural Resources and Regional Planning Chinese Academy of Agricultural Sciences Beijing China; ^2^ Microbial Processes and Interactions Research Unit Gembloux Agro‐Bio Tech University of Liège Gembloux Belgium; ^3^ Laboratory of Quality & Safety Risk Assessment for Microbial Products Ministry of Agriculture Beijing China; ^4^ The Institute of Soil Fertility and Environmental Sources Heilongjiang Academy of Agricultural Sciences Harbin China

**Keywords:** fungal community composition, illumina miseq sequencing, inorganic fertilizer, manure, soil degradation

## Abstract

How fungi respond to long‐term fertilization in Chinese Mollisols as sensitive indicators of soil fertility has received limited attention. To broaden our knowledge, we used high‐throughput pyrosequencing and quantitative PCR to explore the response of soil fungal community to long‐term chemical and organic fertilization strategies. Soils were collected in a 35‐year field experiment with four treatments: no fertilizer, chemical phosphorus, and potassium fertilizer (PK), chemical phosphorus, potassium, and nitrogen fertilizer (NPK), and chemical phosphorus and potassium fertilizer plus manure (MPK). All fertilization differently changed soil properties and fungal community. The MPK application benefited soil acidification alleviation and organic matter accumulation, as well as soybean yield. Moreover, the community richness indices (Chao1 and ACE) were higher under the MPK regimes, indicating the resilience of microbial diversity and stability. With regards to fungal community composition, the phylum Ascomycota was dominant in all samples, followed by Zygomycota, Basidiomycota, Chytridiomycota, and Glomeromycota. At each taxonomic level, the community composition dramatically differed under different fertilization strategies, leading to different soil quality. The NPK application caused a loss of Leotiomycetes but an increase in Eurotiomycetes, which might reduce the plant–fungal symbioses and increase nitrogen losses and greenhouse gas emissions. According to the linear discriminant analysis (LDA) coupled with effect size (LDA score > 3.0), the NPK application significantly increased the abundances of fungal taxa with known pathogenic traits, such as order Chaetothyriales, family *Chaetothyriaceae* and *Pleosporaceae*, and genera *Corynespora*,* Bipolaris,* and *Cyphellophora*. In contrast, these fungi were detected at low levels under the MPK regime. Soil organic matter and pH were the two most important contributors to fungal community composition.

## 1 INTRODUCTION

Mollisols (black soil regions) are widely distributed in northeast China and are considered highly fertile soils. Consequently, these black soil regions have become important agricultural areas for grain production and cultivation (Zhao et al., 2015). However, several decades of excessive cultivation and intensive fertilization have caused substantial loss of soil and soil productivity (Liu et al., 2015; Singh, Verma, Ansari, & Shukla, 2014). Inappropriate chemical fertilizer applications cause serious soil degradation and environmental pollution (Yin et al., 2015), especially the overuse of nitrogen (N) fertilizers, which have a litany of consequences, including climate change, greenhouse gas emission, marine and freshwater eutrophication, soil acidification and soil microbial diversity, activity, and biomass reduction (Edwards, Zak, Kellner, Eisenlord, & Pregitzer, 2011; Guo et al., 2010; Ramirez, Craine, & Fierer, 2012; Zhou et al., 2015). Furthermore, excessive use of N fertilizer can alter the dynamics of plant populations, cause changes in plant species compositions, and increase the concentrations of elements like manganese, iron, and aluminum, which harm plants at a high concentration (Clark et al., 2007; Johnson, Wolf, & Koch, 2003).

The abovementioned problems will be difficult to solve as long as excessive N fertilization inputs continue, thus reductions in chemical fertilizer application have been advocated (Williams, Börjesson, & Hedlund, 2013). Organic amendments can supply N to crops and are beneficial for soil quality, causing residual N effects the year after their application (Schröder, Uenk, & Hilhorst, 2007). Manure, as an important source of organic matter (OM), is an effective substitute for chemical N inputs and its use could solve the problems without decreasing crop yields (Ding et al., 2017). However, we know less about the effects of manure application on soil microorganisms, which are valuable indicators of soil quality and are involved in stabilizing soil structure (Chu et al., 2007; Romaniuk, Giuffré, Costantini, & Nannipieri, 2011). Compared with bacteria, soil fungal diversity is more sensitive to soil fertility (He, Zheng, Chen, He, & Zhang, 2008), due to their organic N and phosphorus (P) acquisition capabilities (Behie & Bidochka, 2014; Näsholm, Kielland, & Ganeteg, 2009) and important roles in nutrient cycling (Cairney, 2011; Szuba, 2015). A stable and appropriate fungal community composition is also beneficial for soil biochemical cycle, and also leads to a healthy and stable surrounding ecosystem for plants (Sun, Liu, Yuan, & Lian, 2016). Thus, it is of crucial interest to investigate soil fungal communities.

In our previous studies, the impacts of long‐term fertilizations on bacterial community composition have been examined (Zhou et al., 2015). Inorganic fertilization led to a significant decrease in the biodiversity and abundance of bacteria, and the influence of more concentrated fertilizer treatments was greater than that of lower concentrations. However, a comprehensive understanding of the fungal responses is still unclear, especially when organic manure is substituted for chemical N fertilizer. In this study, soils were collected during a 35‐year field experiment in the Chinese Mollisols, and high‐throughput pyrosequencing and quantitative PCR (qPCR) technology were performed to analyze soil fungal community composition and abundance. Here, we hypothesize that: (1) the differences in fungal community composition and abundance are a result of long‐term fertilization strategies that induces changes in soil properties; (2) manure helps shift the soil fungal community to a good status, whereas chemical fertilizer applications exhibit the opposite pattern; and (3) the shifts of fungal community may mainly result from changes in the soil pH and OM. In summary, understanding the responses of fungal community composition to different fertilization strategies is not only an effective way to reveal the relationship between intensive fertilization and black soil degradation but is also meaningful for determining appropriate fertilization applications to improve and maintain soil fertility.

## 2 MATERIALS AND METHODS

### 2.1 Field experiments and soil sampling

This study has been performed in an experimental field with a wheat–maize–soybean crop rotation since 1980 in Harbin City, Heilongjiang Province, China (45°40′N, 126°35′E). The climate for this region is characterized as typical temperate monsoon, with an annual mean air temperature of 3.5°C, evaporation of 1,315 mm and precipitation of 533 mm. The field experiment was set up as a block design with three replicates, with each block comprised of a different treatment randomized in plots of 9 × 4 m. Chemical fertilizers were applied as urea (75 kg/hm^2^), calcium superphosphate plus ammonium hydrogen phosphate (150 kg/hm^2^), and potassium sulfate (75 kg/hm^2^), respectively. The horse manure was used at approximately 18,600 kg/hm^2^. More details on the experimental field were shown in our previous study (Wei et al., 2008).

Soils were collected among plant rows after the soybean harvest in September 2014. Four treatments with three replicates were chosen: no fertilizer (CK), chemical P and potassium (K) fertilizer (PK), chemical N, P and K fertilizer (NPK), and chemical P and K fertilizer plus manure (MPK). For each replicate plot in every treatment, six cores were randomly collected in the ploghed soil layer (5–20 cm) after removing plant residues and gravels. Cores were combined and mixed uniformly to obtain a homogeneous blend and subsampled into three parts. One part was reserved at −80°C, and the other two were used as two subsamples. A total of 24 soil subsamples were obtained. Soil chemical properties and molecular analyses were performed for each subsample.

### 2.2 Analyses of soil chemical properties and soybean yield

Soil chemical properties, including soil pH, OM, Total N (TN), nitrate nitrogen (NO_3_^−^–N), ammonium nitrogen (NH_4_^+^–N), Total P (TP), available P (AP), Total K (TK), and available K (AK) were analyzed after being air dried at room temperature and passed through a 2.0‐mm sieve. Soil pH was measured with a pH meter using a 1:1 sample: water extract. Soil OM was assayed by applying the K_2_Cr_2_O_7_‐capacitance method (Strickland & Sollins, 1987). TN was measured using the Kjeldahl method (Huang et al., 2007). NH_4_^+^–N and NO_3_^−^–N were extracted by 2 mol/L KCl solution and subjected to flow injection analysis according to Hart, Stark, Davidson, and Firestone (1994). A modified method of resin extraction was used for the AP analysis (Hedley & Stewart, 1982), and TP was determined using the colorimetric method (Garg & Kaushik, 2005). TK and AK were analyzed by atomic absorption spectrometer and flame photometry, respectively, as recommended by Helmke and Sparks (1996) and Habib, Javid, Saleem, Ehsan, and Ahmad (2014). Soybean yields under different conditions were recorded after harvest.

### 2.3 Total DNA extraction

Total DNA was extracted from 0.25 g soil in each subsample using a MOBIO PowerSoil DNA Isolation Kit (Carlsbad, CA, USA) according to the manufacturers’ protocol with modifications (Fierer et al., 2012). Briefly, six successive replicate extractions were taken from each subsample and fixed together as one DNA template to provide enough total DNA (Zhou et al., 2016). DNA purification followed, and then, DNA concentration and quality (*A*_260_/*A*_280_) of the extracts were estimated visually using a NanoDrop ND‐1000 UVevis spectrophotometer (Thermo Scientific, Rockwood, TN, USA).

### 2.4 qPCR analysis

The soil fungal abundance levels were quantified using the qPCR detection system (Applied Biosystems 7500, CA, USA). The internal transcribed spacer (ITS) primers ITS4F (5′‐TCC TCC GCT TAT TGA TAT GC‐3′) and ITS5 (5′‐GGA AGT AAA AGT CGT AAC AAG G‐3′) were used to amplify the fungal ITS region of ribosomal RNA gene as recommended by Schoch et al. (2012). The components of the reaction mixture (25 μl) and the optimized conditions for amplification were as previously reported (Zhou et al., 2016). The qPCR was carried out with three replicates for each soil subsample. The standard curve was generated using 10‐fold serial dilutions of a plasmid containing the *ITS* gene insert. The abundances of the bacterial *16S rRNA* gene copies were quantified using the same method as for the *ITS* gene, with primers 515F and 806R (Lauber, Ramirez, Aanderud, Lennon, & Fierer, 2013), and presented in Table S1. The value of the fungi/bacteria ratio (*F*/*B* ratio) was calculated by dividing the *ITS* gene copy number by the *16S rRNA* gene copy number (Wurzbacher, Rösel, Rychła, & Grossart, 2014).

### 2.5 Illumina MiSeq sequencing

The fungal ITS1 region was amplified using the primers ITS1F (5′‐CTT GGT CAT TTA GAG GAA GTA A‐3′) and ITS2 (5′‐GCT GCG TTC TTC ATC GAT GC‐3′) as previously documented (Buee et al., 2009; Degnan & Ochman, 2012; Ding et al., 2017). The ITS1F/ITS2 primers are considered as the universal DNA barcode markers for the molecular identification of fungi (Blaalid et al., 2013; Schoch et al., 2012). Barcodes were connected with primers and were used to separate raw data, allowing multiple samples to be pooled into one run of Illumina MiSeq sequencing. The conditions of the PCR reaction were as follows: 94°C for 2 min; 32 cycles of 94°C for 30 s, 55°C for 30 s and 72°C for 30 s; and 72°C for 5 min. PCR products were mixed (equimolar ratio) after purification, creating a DNA pool. Sequencing libraries were generated. Finally, the libraries were sequenced on Illumina MiSeq platform at Personal Biotechnology Co., Ltd. (Shanghai, China).

### 2.6 Data processing and statistical analyses

Barcode sequences were removed according to the methods of Edgar, Haas, Clemente, Quince, and Knight (2011). The raw sequence reads were processed using QIIME (version 1.7.0, http://qiime.org/) (Caporaso et al., 2010) and referring to the default parameters to obtain valid tags (Bokulich et al., 2013). Singletons, non‐bacterial and non‐fungal OTUs were removed, and the OTU abundance levels were normalized based on the sample with the least number of sequences. To perform a fair comparison between samples, all subsequent analyses were performed according to the normalized data (Zhou et al., 2016). Then, operational taxonomic units defined by clustering at the 97% similarity level were generated and taxonomically classified using a BLAST algorithm against the UNITE database release 5.0 (Koljalg et al., 2014) with a minimal 80% confidence estimate (Bokulich & Mills, 2013). The UNITE and INSDC fungal ITS databases were used as references for classification (Abarenkov et al., 2010). The sequences were uploaded and deposited in the National Center for Biotechnology Information (NCBI) Sequence Read Archive (SRA) under the accession number SRP092759.

The fungal α‐diversity index (including Shannon, Simpson, Chao1, and ACE) was analyzed using Mothur software (version 1.31.2, http://www.mothur.org/) (Schoch et al., 2012). The unweighted Fast UniFrac metric was calculated to construct distance matrices using QIIME (Caporaso et al., 2010). A principal coordinate analysis (PCoA) based on the unweighted Fast UniFrac metric was carried out to compare between‐sample variations in fungal community composition (Marsh, O Sullivan, Hill, Ross, & Cotter, 2013). A linear discriminant analysis coupled with effect size (LEfSe) was performed to distinguish significantly different fungal taxa between MPK and NPK regimes to the genus or higher taxonomy level (Segata et al., 2011). The software of CANOCO 5.0 was used for ribosomal database project (RDP) analysis with a minimal 60% threshold to explore possible linkages between fungal community and soil property, followed the method of Braak and Smilauer (2012). An analysis of variance was performed on all experimental data using SPSS (v.19). In all tests, a *p‐*value <.05 was considered statistically significant.

## 3 RESULTS

### 3.1 Soil properties and soybean yields under different fertilization regimes

Soil properties under different fertilization regimes are shown in Table 1. PK and NPK applications significantly decreased soil pH, whereas the MPK application alleviated soil acidification. The MPK application also had an accumulative effect on soil OM. Compared with the CK, the three fertilization strategies significantly increased the concentrations of AK and TK, as well as AP and TP. The NPK application significantly increased the TN concentration, whereas the concentrations of NO_3_^−^–N and NH_4_^+^–N were lower than under the MPK regime. In addition, soybean yields were significantly higher under the fertilization regimes, with the MPK application being the most effective strategy (2702 kg·ha^−1^).

**Table 1:** Soil properties and soybean yield under different fertilization regimes

Fertilization regimes	pH	OM (g·kg^−1^)	AK (g·kg^−1^)	TK (g·kg^−1^)	NH_4_^+^ (mg·kg^−1^)	NO_3_^−^ (mg·kg^−1^)	TN (g·kg^−1^)	AP (g·kg^−1^)	TP (g·kg^−1^)	Soybean yield (kg·ha^−1^)
CK	6.43 ± 0.08c	24.39 ± 0.37a	0.17 ± 0.03a	6.30 ± 0.89a	35.01 ± 1.16a	2.45 ± 0.88a	1.20 ± 0.05a	0.02 ± 0.01a	0.44 ± 0.03a	1812.67 ± 141.99a
PK	6.18 ± 0.04b	25.51 ± 0.30c	0.24 ± 0.01b	28.57 ± 2.25b	37.80 ± 2.95a	3.62 ± 0.57b	1.26 ± 0.03a	0.89 ± 0.04c	0.73 ± 0.02c	2377.33 ± 118.85bc
NPK	5.54 ± 0.04a	24.88 ± 0.25b	0.23 ± 0.03b	30.36 ± 1.02b	37.30 ± 6.29a	4.53 ± 0.91bc	1.43 ± 0.08b	0.94 ± 0.06d	0.70 ± 0.02bc	2241.33 ± 186.11b
MPK	6.38 ± 0.05c	27.47 ± 0.41d	0.23 ± 0.03b	28.43 ± 3.93b	39.36 ± 6.95a	5.17 ± 0.67c	1.20 ± 0.03a	0.66 ± 0.01b	0.61 ± 0.05b	2702.67 ± 169.39c

Values are means ± standard deviations (*n* = 6). Values within the same column followed by different letters indicate significant differences (*p* < .05) according to Tukey's multiple comparison.

Fertilization regimes: CK, no fertilizer; PK, chemical phosphorus and potassium fertilizer; NPK, chemical phosphorus, potassium, and nitrogen fertilizer; MPK, chemical phosphorus and potassium fertilizer plus manure.

Soil properties: AP, available phosphorus; AK, available potassium; NH_4_^+^, ammonium nitrogen; NO_3_^−^, nitrate nitrogen; TK, total potassium; TP, total phosphorus; TN, total nitrogen; OM, organic matter.

### 3.2 Fungal ITS gene copy number under different fertilization regimes

**Figure 1:**
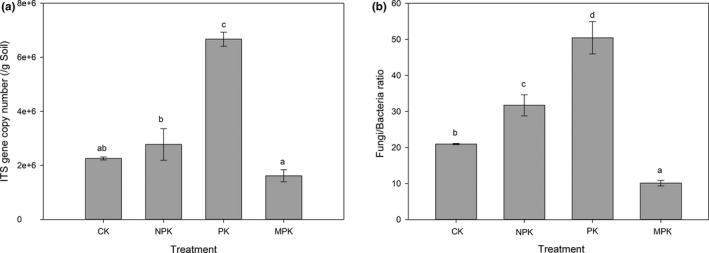
Results of the quantitative PCR. (a) The abundance of fungi as indicated by the number of *ITS* copies; (b) Fungi/Bacteria ratios under different fertilization regimes. Same letters above columns indicate no significant difference (*p* *<* .05, Tukey's test)

The values of the fungal *ITS* copy number ranged from 1.62 × 10^6^ to 6.67 × 10^6^ g^−1^ soil with significant differences (Figure 1a). Compared with the CK, PK, and NPK applications increased the *ITS* gene copies, resulting in a significant increase in the *F*/*B* ratio, whereas MPK applications exhibited the opposite pattern (Figure 1b). In addition, there were significant positive correlations between *ITS* gene copy number and TP (*r* = .613, *p* < .01) and AP (*r* = .435, *p* < .05), referring to Pearson's correlations (Table S2). Moreover, the *F*/*B* ratio showed significantly negative correlations with soil pH (*r* = −.912, *p* < .01) and OM (*r* = −.572, *p* < .01), but was positively correlated with TN (*r* = .795, *p* < .01) and TP (*r* = .523, *p* < .01).

### 3.3 Fungal diversity analysis under different fertilization regimes

A total of 1,399,128 raw sequence reads were obtained from the Illumina MiSeq platform analysis of 24 soil subsamples, and 1,179,936 effective sequences were produced after processing. The high‐quality percentage was more than 82%, with a mean read length of 280 bp. More statistical data of sequencing in different samples are detailed in Table S3. Rarefaction analysis (Figure S1) displayed similar trends whereby the greatest OTU occurred under the MPK regime, and the lowest value occurred in the NPK treatment. The Good's coverage values (0.991–0.992) indicated that there were sufficient reads to obtain the fungal diversity. With regards to fungal community richness indices (Table 2), PK and NPK applications reduced Chao1 and ACE indices, whereas the MPK application led to the greatest indices. In addition, Pearson's correlations (Table S2) showed that the Chao1 index was significantly positively correlated with OM (*r* = .564, *p* < .01).

**Table 2:** Estimated numbers of observed operational taxonomic units (97% similarity) and diversity of soil in different fertilization regimes

Fertilization regimes	Observed species	Chao1	Ace	Simpson	Shannon	Goods coverage
CK	895.83 ± 59.44a	1050.1 ± 46.2a	1084.1 ± 80.8ab	0.979 ± 0.016a	7.22 ± 0.27ab	0.992 ± 0.0015a
PK	877.33 ± 97.73a	1028.6 ± 37.9a	1040.9 ± 57.8a	0.987 ± 0.002a	7.46 ± 0.21b	0.992 ± 0.0019a
NPK	812.00 ± 40.87a	1034.1 ± 54.5a	1049.0 ± 41.9a	0.985 ± 0.003a	7.19 ± 0.14a	0.992 ± 0.0020a
MPK	914.17 ± 167.33a	1140.2 ± 101.7b	1164.8 ± 101.6b	0.986 ± 0.004a	7.40 ± 0.14ab	0.991 ± 0.0038a

Values within the same column followed by different letters indicate significant differences (*p* < .05) according to Tukey's multiple comparison.

**Figure 2:**
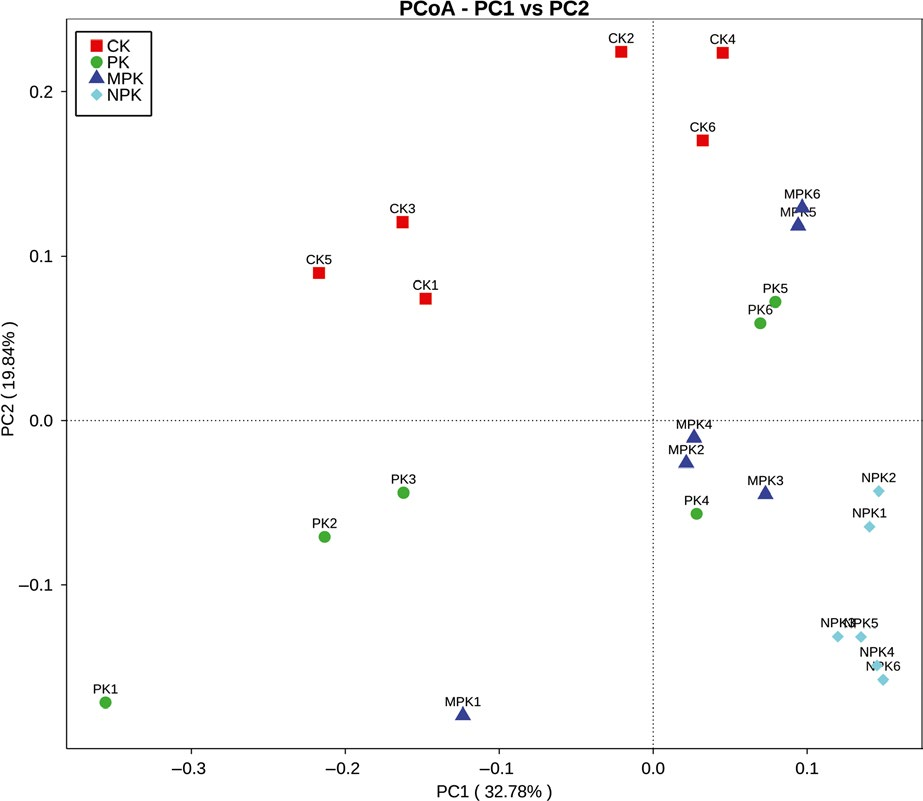
PCoA of the pyrosequencing reads based on the unweighted Fast UniFrac metric

A PCoA was performed to analyze the impacts of fertilization strategies on fungal community structure (Figure 2). The two axes, PC1 and PC2, explained 32.78% and 19.84% of the total variation, respectively. The NPK plots located in the lower right corner and were far from the CK; whereas PK and MPK plots were clustered together and located in the middle. Compared with the CK, long‐term fertilization strategies clearly changed the fungal community composition due to the effects of chemical N inputs.

### 3.4 Fungal community compositions and relative abundance under different fertilization regimes

**Figure 3:**
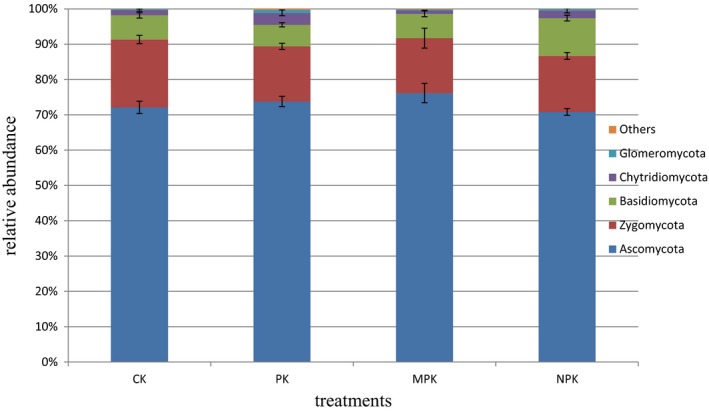
Relative abundance of phylogenetic phyla under different fertilization regimes

**Figure 4:**
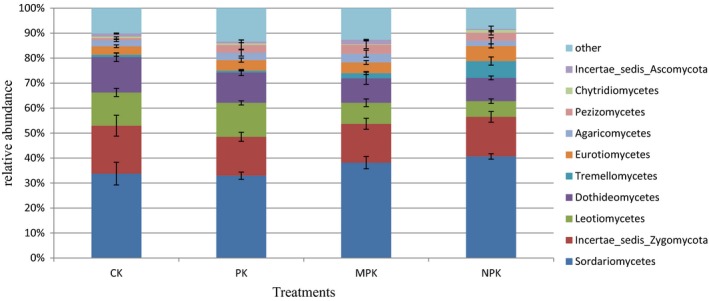
Relative abundance of phylogenetic classes under different fertilization regimes. At least one group's relative abundance is more than 0.1% of the total sequences

**Figure 5:**
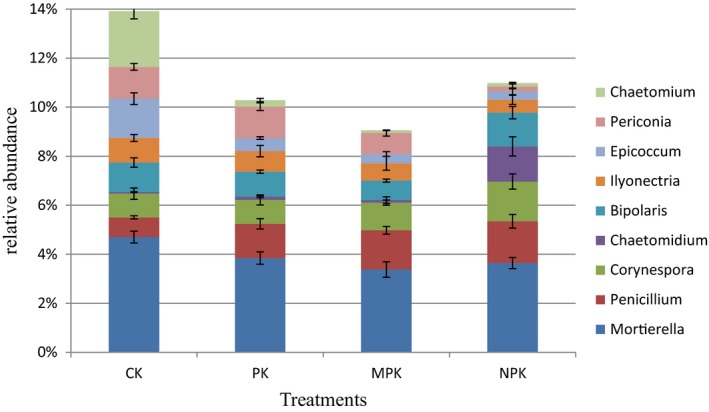
Relative abundance of phylogenetic genera under different fertilization regimes. At least one group's relative abundance is more than 1% of the total sequences

Phyla Ascomycota, representing 70.83%–76.16% of the total sequences, was dominant, followed by Zygomycota (15.56%–19.22%), Basidiomycota (6.14%–10.72%), Chytridiomycota (0.94%–3.37%), and Glomeromycota (0.28%–0.83%) (Figure 3). Compared with the CK, the MPK application significantly increased the relative abundance of the phyla Ascomycota, which decreased under the NPK regime. Sordariomycetes was dominant at class level, followed by Incertae_sedis_Zygomycota, Leotiomycetes, and Dothideomycetes (shown in Figure 4, at least one group with a relative abundance >0.1%). NPK and MPK applications significantly increased the relative abundance of Sordariomycetes, but decreased those of Leotiomycetes and Dothideomycetes. The abundances of the classes Eurotiomycetes and Tremellomycetes were significantly higher under the NPK regime than under the others. All the fertilization treatments had positive effects on the Pezizomycetes. At the genus level (Figure 5), all the fertilization strategies significantly decreased the relative abundances of *Mortierella*,* Chaetomium,* and *Epicoccum*, but *Penicillium* was increased. *Periconia* and *Ilyonectria* were lower under the NPK and MPK regimes. In particular, the chemical N fertilizer significantly increased the abundances of *Chaetomidium* and *Corynespora*.

### 3.5 Significantly different fungal taxa occurred under the NPK and MPK regimes

**Figure 6:**
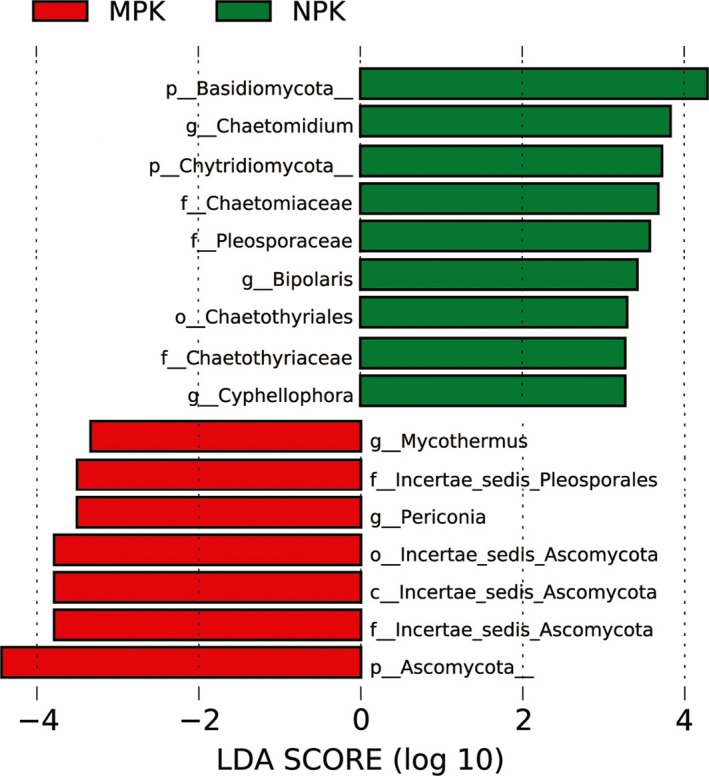
Histogram of the linear discriminant analysis scores computed for features differentially abundant between NPK and MPK samples identified by LEfSe (LDA score > 3)

The LEfSe analysis distinguished the presence of significantly different fungal taxa under the NPK and MPK regimes (average relative abundance > 0.01; Figure 6). The linear discriminant analysis score was greater than 3.0. The MPK‐treated samples had significantly higher abundance of the phylum Ascomycota, and genera *Mycothermus* and *Periconia*, whereas the phyla Basidiomycota and Chytridiomycota, the order *Chaetothyriales*, the families *Chaetomiaceae*,* Pleosporaceae,* and *Chaetothyriaceae*, and genera *Chaetomidium*,* Bipolaris,* and *Cyphellophora* were overrepresented under the NPK regime.

### 3.6 Correlation between fungal community composition and soil properties

**Figure 7:**
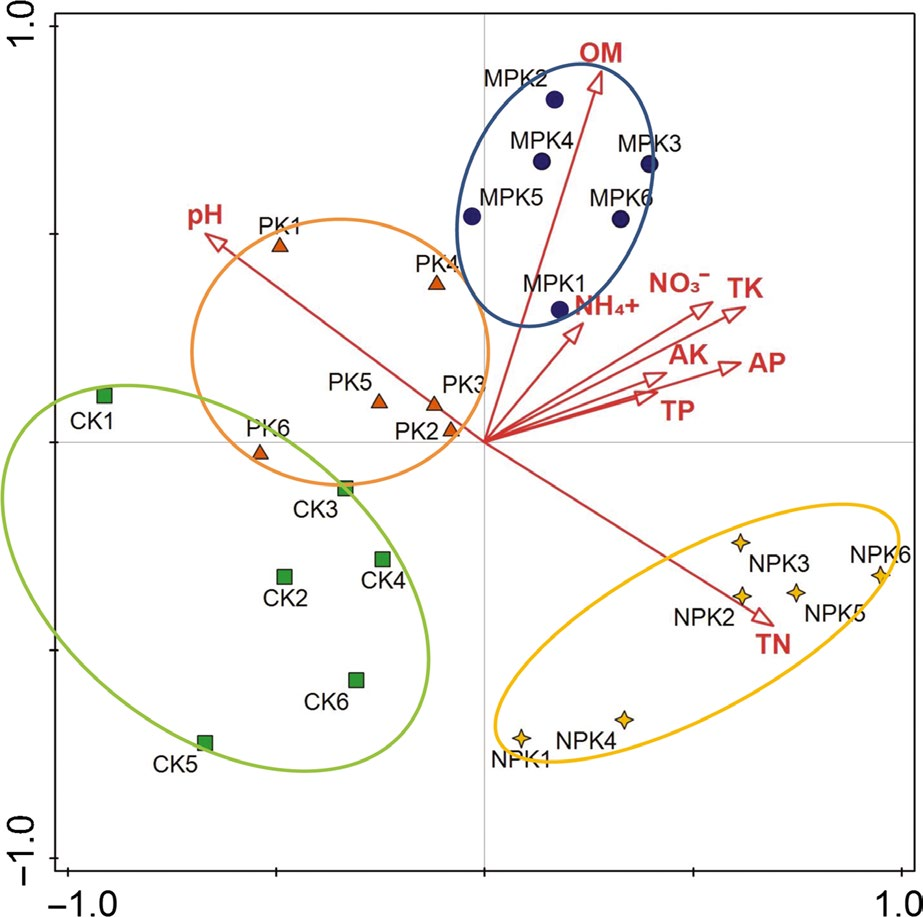
Redundancy analysis of soil bacterial communities and soil characteristics for individual samples. Soil factors indicated in red text include available phosphorus (AP), available potassium (AK), pH, soil concentration of NH_4_^+^ (NH_4_^+^), soil concentration of NO_3_^−^ (NO_3_^−^),total nitrogen (TN), total potassium (TK), total phosphorus (TP), and organic matter (OM)

Based on the redundancy analysis (Figure 7), all the selected soil properties accounted for 56.8% of the explanatory variables in the fungal community composition among the samples. The primary contributors in shifting the fungal community were soil OM (*F* = 4.5, *p =* .002) and pH (*F* = 4.1, *p* = .002), which individually accounted for 14.9% and 15.7% of the variation, respectively. The other soil properties affected fungal community composition in the following order: AK > AP > TN > TP > NO_3_^−^–N = NH_4_^+^–N > TK. In addition, the plots of CK, PK, NPK and MPK were well grouped and separated from the NPK plot.

Pearson's correlations (Table S4) showed that Ascomycota was positively correlated with OM (*r* = .709, *p* < .01) and pH (*r* = .508, *p* < .05), whereas Zygomycota was negatively correlated with AP, AK, OM, TK, TP, and NO_3_^−^–N (*p* < .01). Soil pH had negative impacts on Basidiomycota; however, the effect of TN was positive. Both AP and TP were positively correlated (*p* < .01) with Chytridiomycota and Glomeromycota.

## 4 DISCUSSION

### 4.1 Improvements in soil acidification, OM accumulation, and soybean yield

Long‐term chemical fertilizer applications, especially the NPK application, significantly increased soil acidification; however, manure could effectively alleviate soil acidification, perhaps due to the buffering functions of organic acids, carbonates, and bicarbonates (García‐Gil, Ceppi, Velasco, Polo, & Senesi, 2004; Whalen, Chang, Clayton, & Carefoot, 2000). Furthermore, manure had macronutrient status, contributing to the significant accumulation of soil OM (Xie et al., 2014). In turn, the high productivity resulting from organic manure increases the amounts of OM in the soil, in the form of root exudates, decaying roots and aboveground residues, which are beneficial for soil OM accumulation (Geisseler & Scow, 2014). In addition, soybean yields were significantly higher under the fertilization regimes, with the MPK application being the most effective fertilization strategy. The results agreed well with other findings (Zhao et al., 2014).

### 4.2 Changes in the ITS gene's abundance and *F*/*B* ratio

Compared with the CK, *ITS* gene copies were increased under both PK and NPK regimes, which confirmed the positive stimulatory effects of chemical fertilizer on fungal populations (Zhou et al., 2016). Moreover, Pearson's correlations showed positive correlations between fungal abundance and AP (*r* = .435, *p* < .05) and TP (*r* = .613, *p* < .01), which were quite similar to other findings (Kuramae et al., 2012).

In addition, the *F*/*B* ratio was considered an indicator of ecosystem processes, as the changes in ratio were likely to be related to decomposition, nutrient cycling, C‐sequestration potential, and ecosystem self‐regulation (Strickland & Rousk, 2010). In this study, the *F*/*B* ratios under CK and MPK regimes were lower than those of PK and NPK, probably due to the acidification of soil induced by chemical inputs. As documented by Joergensen and Wichern (2008) and Rousk, Brookes, and Bååth (2009), fungi have been found to be more acid tolerant than bacteria leading to increased fungal dominance in acidic soils. Moreover, the *F*/*B* ratio was highest under the PK regime probably due to the better adaptability of fungal species to N limitation compared with bacteria (Rousk & Frey, 2015). The *F*/*B* ratio under the MPK regime was significantly lower than others, indicating a higher turnover rate of easily available substrates (Rousk, Brookes, & Bååth, 2010) and highly productive crop soils (Strickland & Rousk, 2010). Additionally, the *F*/*B* ratio was significantly positive correlated with soil pH (*r* = .648, *p* < .01), this might be due to the different responses of bacteria and fungi to lower pH levels, namely the significant suppressive effect of bacteria and well tolerance of fungi (Coyne, 1999; Rousk, Bååth, et al., 2010).

### 4.3 Effects on fungal α‐diversity

Microbial diversity in soil was closely related to soil quality and the nutrient cycling rate (Nevarez et al., 2009). The richer the biodiversity, the more stable the soil (Chaer, Fernandes, Myrold, & Bottomley, 2009). The lower biodiversity of fungi also caused unsustainable crop production and an unstable ecosystem (Maček et al., 2011). In this study, PK and NPK applications reduced the fungal community richness indices (Chao1 and ACE), whereas the MPK application significantly increased them. This might be explained by the complex organic compounds present in manure requiring various microorganisms to degrade. The results confirmed previous findings that a high microbial diversity was always found under organic amendment regimes rather than chemical regimes (Esperschütz, Gattinger, Mäder, Schloter, & Fließbach, 2007). Compared with soil nutrients, the Chao1 index was positively correlated with soil OM (*r* = .564, *p* < .01), which probably provided macronutrient for fungi and stimulated the microbial biomass and diversity (Peacock et al., 2001). In conclusion, the substitution of chemical N fertilizer with organic manure, which is beneficial for the resilience of microbial diversity (Naeem & Li, 1997) and soil productivity (Sapp, Harrison, Hany, Charlton, & Thwaites, 2015), was a good way to reduce anthropogenic N inputs.

### 4.4 Impact on fungal community composition

The phylum Ascomycota was dominant in all the fertilization regimes. Similar results have been observed in other studies (Xiong et al., 2014; Li, Ding, Zhang, & Wang, 2014). The abundance of Ascomycota under the NPK regime was lower than under the PK and MPK regimes, which contrasted with other findings that Ascomycota was enhanced by relatively high N inputs (Nemergut et al., 2008; Paungfoo‐Lonhienne et al., 2015). This could be explained by the fact that members of the Ascomycota are adapted to the appropriate N content (Klaubauf et al., 2010) but were vulnerable to excess N levels (Wang et al., 2015).

At the class level, Sordariomycetes was the most dominant member, in line with other findings (Zhou et al., 2016). Compared with the CK and PK application, the relative abundances of Sordariomycetes were significantly higher under the NPK and MPK regimes, probably due to sufficient nutrients in soil (Ding et al., 2017). However, Dothideomycetes showed the opposite pattern, namely, they were significantly lower under the NPK and MPK regimes, indicating positive effects on soil quality, as many of the taxa appeared to be plant pathogens (Lyons, Newell, Buchan, & Moran, 2003). Leotiomycetes dominance was lowest under the NPK regime, indicating a negative correlation with the chemical N input (Freedman, Romanowicz, Upchurch, & Zak, 2015; Zhou et al., 2016). The decline of Leotiomycetes under NPK regime probably caused a loss of plant–fungal symbioses under high N input conditions (Dean et al., 2014). Additionally, the NPK application produced a higher abundance of Tremellomycetes, which probably benefited inorganic matter decay (Freedman et al., 2015). The abundance of Eurotiomycetes was also higher under the NPK regime, probably causing N loss in the soil and greenhouse gas emissions due to its N_2_O‐producing activity (Jasrotia et al., 2014; Mothapo, Chen, Cubeta, Grossman, & Fuller, 2015). The abundances of Pezizomycetes were significantly high under all the fertilization regimes, which may be the result of soil OM accumulation due to decaying wood, dung, leaf litter, and twigs (Stajich, 2015).

A thorough investigation at the genus or higher taxonomic level showed differences among the treatments. More harmful fungal taxa with known pathogenic traits were also overrepresented under the NPK regime, such as the order Chaetothyriales, families *Chaetothyriaceae*,* Pleosporaceae,* and *Chaetomiaceae*, and genera *Corynespora, Bipolaris,* and *Cyphellophora Chaetomidium*. The order Chaetothyriales, family *Pleosporaceae* and genus *Chaetomidium* are well‐known for their animal and human opportunistic pathogens (Arzanlou, Khodaei, & Saadati Bezdi, 2012; Sajeewa et al., 2015; Winka, Eriksson, & Bång, 1998). And family *Chaetomiaceae* includes numerous soil‐born, saprotrophic, endophytic, and pathogenic fungi (Zámocky et al., 2016), and also, several *Cyphellophora* species have also been associated with potential pathogens (Decock, Delgado‐Rodríguez, Buchet, & Seng, 2003). Moreover, some isolates within *Corynespora* are pathogenic to a wide range of hosts (Dixon, Schlub, Pernezny, & Datnoff, 2009) and *Bipolaris* causes significant yield losses as a foliar disease constraint (Road, 2002). Obviously, long‐term NPK applications may induce the incidence rates of fungal diseases. In contrast, these fungi were detected at low levels under the MPK regime. Meanwhile, the genus *Mycothermus* was also significantly more dominant under the MPK regime, which benefits the decomposition of cellulose because of its appreciable titers of cellulases and hemicellulases (Basotra, Kaur, Di Falco, Tsang, & Chadha, 2016). Thus, manure helps shift the soil fungal community to a good status, whereas chemical fertilizer applications exhibit the opposite pattern.

### 4.5 Soil properties effects on fungal community composition

In line with previous findings (Liu et al., 2015; Ding et al., 2017), we concluded that soil OM and pH were the most important contributors to the variation in the fungal community composition, based on the redundancy analysis. As documented by Broeckling, Broz, Bergelson, Manter, and Vivanco (2008), the majority of fungi are heterotrophs and depend on exogenous C for growth, thus labile OM has a profound influence on their abundance. Moreover, soil pH also played a key role in shaping fungal community composition (Ding et al., 2017; Kim et al., 2015). This could be explained by the more sensitivity of fungi to a pH change (Liu et al., 2015). Additionally, soil pH may affect fungal community composition by responding to other variables and may provide an integrated index of soil conditions. Hydrogen ion concentration varies by many orders of magnitude across various soils and, as numerous soil properties are related to soil pH, these factors may have driven the observed shifts in community composition (Rousk, Bååth, et al., 2010; Shen et al., 2013; Xiong et al., 2012). Thus, soil microorganisms could rapidly respond to the changes in the environmental conditions (Eilers, Debenport, Anderson, & Fierer, 2012), such as soil chemical or physical properties induced by fertilization. In turn, shifts in microorganism composition could influence soil quality and plant growth.

In addition, long‐term different fertilization strategies had significant effects on bacterial β‐diversity and shaped variant microbial compositions in the soil (Zhou et al., 2017). In this study, a PCoA revealed the relationship between soil fertilization and the fungal community. The NPK plot, located in the lower right corner, was far from the CK plot, indicating a strong effect of chemical fertilizer; however, the PK and MPK plots were clustered together and near the CK plot, suggesting the effective resilience of organic manure on fungal community structure, in line with Ding et al. (2017).

## 5 CONCLUSION

Our findings determined the responses of soil fungal community composition to long‐term fertilization strategies in black soil. Such shifts may mainly be derived from changes in the soil pH and OM. Compared with chemical fertilization, manure applications alleviated soil acidification, accumulated soil OM, increased soil nutrients, improved soil fungal community composition, and restored soil microbial alterations, leading to improvements of soil quality and soybean yield. The results highlighted the potential of organic manure as a substitute for chemical N fertilizers in the sustainable development of Chinese Mollisols.

## CONFLICT OF INTEREST

No conflict of interest is declared.
