# Supplementary figures and images for: Responses of fungal community composition to long‐term chemical and organic fertilization strategies in Chinese Mollisols
Source: Microbiologyopen. 2018 Mar 23;7(5):e00597. doi: 10.1002/mbo3.597 (PMC6182557; doi:10.1002/mbo3.597)

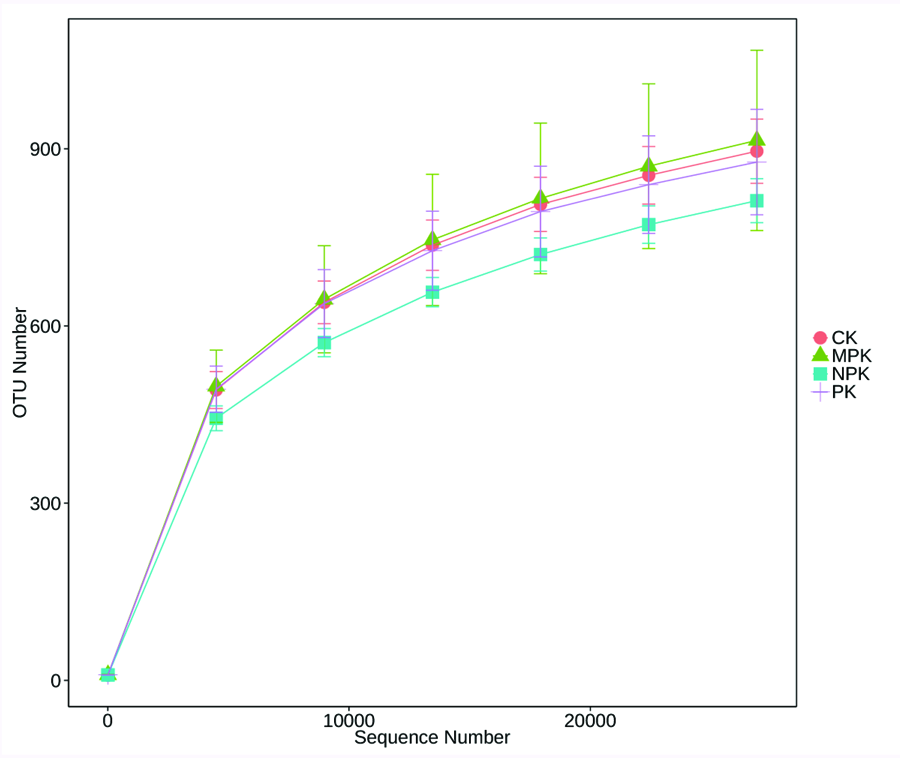

Supplement: Supplementary file 1 [file MBO3-7-e00597-s001.tif]
