# Supplementary material for: Responses of fungal community composition to long‐term chemical and organic fertilization strategies in Chinese Mollisols
Source: Microbiologyopen. 2018 Mar 23;7(5):e00597. doi: 10.1002/mbo3.597 (PMC6182557; doi:10.1002/mbo3.597)
Supplement: Supplementary file 2 [file MBO3-7-e00597-s002.docx]

Table S1. Number of 16S rDNA copies under different fertilization regimes.

| Fertilization regimes | Number of 16S rDNA copies (10^8^) |
| --- | --- |
| CK | 108 ± 1.64 ab |
| NPK | 86.9 ± 10.2 a |
| PK | 133 ± 17.0 bc |
| MPK | 159 ± 11.1 c |

Values within the same column followed by different letters indicate significant differences (*p <* 0.05) according to Tukey’s multiple comparison.

Table S2 Pearson’s correlations between *ITS* gene copy numbers, F/B ratio, Chao1, and soil properties

| Item | pH | AP | AK | NH_4_^+^ | OM | TK | TP | TN | NO_3_^-^ |
| --- | --- | --- | --- | --- | --- | --- | --- | --- | --- |
| ITS gene copy numbers | -0.090 | **0.435*** | 0.329 | 0.002 | -0.194 | 0.294 | **0.613**** | 0.088 | -0.201 |
| F/B ratio | **-0.912^**^** | 0.523**^**^** | 0.259 | -0.094 | **-0.572**** | 0.331 | 0.508***** | **0.795**** | 0.039 |
| Chao1 | 0.297 | -0.060 | -0.012 | 0.253 | **0.564**** | 0.095 | -0.121 | -0.261 | 0.354 |

**, Correlation is significant at the 0.01 level.

*, Correlation is significant at the 0.05 level.

Table S3 Statistical data of sequencing under different samples

| Sample Name | Raw PE | Nochime | AvgLen | Effective (%) |
| --- | --- | --- | --- | --- |
| CK1 | 71,207 | 61,780 | 280 | 86.76 |
| CK2 | 44,426 | 35,569 | 277 | 80.06 |
| CK3 | 78,895 | 65,497 | 282 | 83.02 |
| CK4 | 44,432 | 35,707 | 277 | 80.36 |
| CK5 | 90,129 | 77,656 | 281 | 86.16 |
| CK6 | 48,228 | 38,882 | 278 | 80.62 |
| PK1 | 41,440 | 35,988 | 288 | 86.84 |
| PK2 | 54,729 | 47,061 | 286 | 85.99 |
| PK3 | 73,041 | 60,877 | 284 | 83.35 |
| PK4 | 54,096 | 44,565 | 278 | 82.38 |
| PK5 | 36,897 | 29,674 | 276 | 80.42 |
| PK6 | 39,057 | 32,802 | 277 | 83.98 |
| MPK1 | 68,584 | 55,157 | 278 | 80.42 |
| MPK2 | 64,025 | 53,622 | 280 | 83.75 |
| MPK3 | 65,573 | 53,462 | 280 | 81.53 |
| MPK4 | 57,183 | 46,807 | 280 | 81.85 |
| MPK5 | 44,850 | 38,416 | 277 | 85.65 |
| MPK6 | 31,502 | 27,222 | 281 | 86.41 |
| NPK1 | 45,056 | 35,228 | 276 | 78.19 |
| NPK2 | 64,193 | 50,783 | 276 | 79.11 |
| NPK3 | 67,225 | 54,328 | 279 | 80.82 |
| NPK4 | 67,956 | 53,854 | 277 | 79.25 |
| NPK5 | 83,104 | 64,275 | 279 | 77.34 |
| NPK6 | 63,292 | 49,164 | 279 | 77.68 |

Table S4. The Pearson’s correlations between relative abundance at the phyla level and soil properties

|  | pH | AP | AK | NH_4_^+^ | OM | TK | TP | TN | NO_3_^-^ |
| --- | --- | --- | --- | --- | --- | --- | --- | --- | --- |
| Ascomycota | **0.508^*^** | 0.040 | 0.199 | 0.142 | **0.709**** | 0.140 | -0.018 | -0.398 | 0.383 |
| Zygomycota | 0.274 | **-0.674**** | **-0.570**** | -0.174 | **-0.493*** | **-0.641**** | **-0.547**** | -0.316 | **-0.572**** |
| Basidiomycota | **-0.831**** | 0.375 | 0.141 | -0.004 | -0.244 | 0.314 | 0.214 | **0.734^**^** | 0.236 |
| Chytridiomycota | -0.292 | **0.447*** | 0.295 | -0.027 | -0.319 | 0.278 | **0.584**** | 0.278 | -0.204 |
| Glomeromycota | -0.198 | **0.563**** | 0.421^*^ | 0.030 | -0.083 | 0.402 | **0.694**** | 0.229 | -0.052 |

**, Correlation is significant at the 0.01 level.

*, Correlation is significant at the 0.05 level.
